# Recent Progress on Hydrogen Production from Ammonia Decomposition: Technical Roadmap and Catalytic Mechanism

**DOI:** 10.3390/molecules28135245

**Published:** 2023-07-06

**Authors:** Xiangyong Huang, Ke Lei, Yan Mi, Wenjian Fang, Xiaochuan Li

**Affiliations:** 1 School of Energy and Environment, Anhui University of Technology, Ma’anshan 243002, China; 2 School of Electrical and Energy Power Engineering, Yangzhou University, Yangzhou 225002, China

**Keywords:** ammonia decomposition, hydrogen production, thermocatalytic, non-thermal plasma-catalytic, electrocatalytic, photocatalytic

## Abstract

Ammonia decomposition has attracted significant attention in recent years due to its ability to produce hydrogen without emitting carbon dioxide and the ease of ammonia storage. This paper reviews the recent developments in ammonia decomposition technologies for hydrogen production, focusing on the latest advances in catalytic materials and catalyst design, as well as the research progress in the catalytic reaction mechanism. Additionally, the paper discusses the advantages and disadvantages of each method and the importance of finding non-precious metals to reduce costs and improve efficiency. Overall, this paper provides a valuable reference for further research on ammonia decomposition for hydrogen production.

## 1. Introduction

Reducing carbon emissions is a pressing global challenge. The development of clean and renewable sources of energy, such as wind or solar, is an effective way. At the same time, efficient and reliable energy storage and carriers are indispensable due to the intermittency and variability of such natural energy sources. Hydrogen is considered an excellent energy carrier due to its carbon-free, renewable, and environmentally friendly features [1,2,3,4]. However, low volume energy density and serious safety issues concerning storage and transportation hinder its large-scale applications. A feasible approach is reversibly storing hydrogen in liquid or solid hydrides (hydrogen carriers) and then generating hydrogen through catalytic processes as required. Among potential hydrogen storage intermediates, ammonia (NH_3_) is the most promising candidate due to its competitive advantages in terms of hydrogen storage capacity, zero carbon emissions, availability, cost, and safety [5,6,7,8].

Ammonias’s hydrogen content is as high as 17.6 wt%, and the hydrogen volume density is about 123 kg-H_2_/m^3^, much higher than other modern hydrogen storage systems [5]. Moreover, ammonia is readily liquefied at room temperature (25 °C) and a light pressure of ca.10atm, resulting in a higher volumetric energy density than pressurized H_2_ [6,7]. In addition, ammonia is widely used as an important chemical material in modern industries, such as nitrogen fertilizers, refrigerants, and NOx-reducing agents. Due to the strong, long-term demand, the infrastructure for its production, storage, and supply has been well developed. As such, compared to other zero-carbon fuels, ammonia is a widely available, practical, and affordable choice [8]. Furthermore, the extensive use of ammonia over the years has contributed to developing safety codes and standards. Thus, ammonia safety has been ensured.

**Figure 1:**
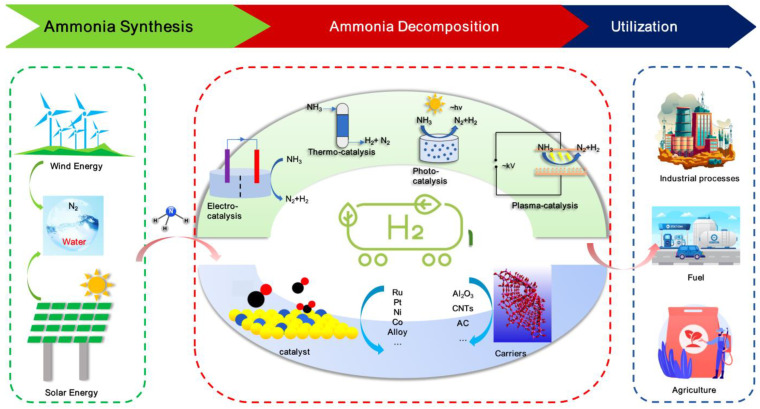
The status of hydrogen production from ammonia decomposition in ammonia economy.

Ammonia decomposition plays a crucial role in the renewable energy sources to hydrogen user’s process, as outlined in Figure 1. The development of these technologies is not synchronized. The production capacity of hydrogen and ammonia is not a significant obstacle to meeting the high demand, while the current ammonia decomposition system does not allow mass hydrogen production with adequate purity. Therefore, ammonia decomposition may become the bottleneck of the hydrogen chain, which is why it is getting dramatically increased attention [9,10,11].

In the past ten years, and especially in the past five, pioneering research and follow-up studies have resulted in new conceptions, methods, and materials for ammonia decomposition. These approaches and underlying mechanisms urgently need to be summarized and understood in detail and are reviewed herein. This paper first analyzes the benefits and drawbacks of various technologies and then describes their characteristics, principles, and mechanisms. Afterward, the composition, performance, and synthesis methods of a series of catalysts are covered.

## 2. Categories of NH_3_ Decomposition

The utilization of NH_3_ as a carrier for H_2_ has become more widespread due to its beneficial properties and importance in industrial settings, as well as its ability to mitigate air pollution [2,12]. Compared to H_2_, NH_3_ is more easily liquefied and stored, making it a more feasible alternative.

$${NH}_{3}\left(g\right)\to \frac{1}{2}N_{2}\left(g\right)+\frac{3}{2}H_{2}\left(g\right)∆H=+46\;kJ/mol$$

**Figure 2:**
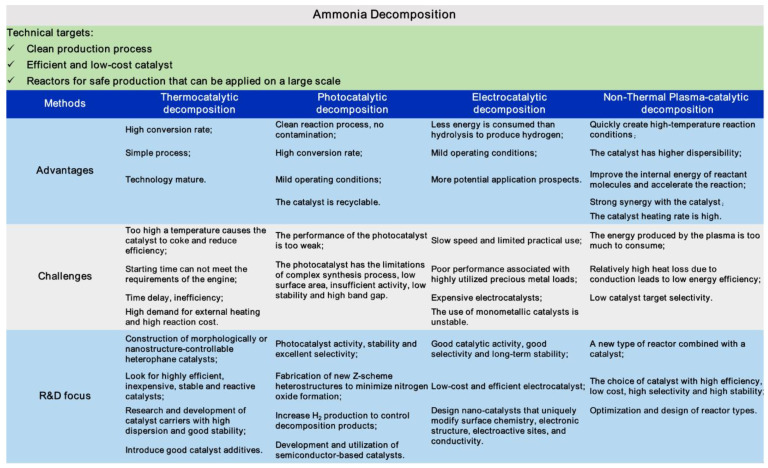
Advantages and disadvantages of different NH_3_ decomposition methods.

**Figure 3:**
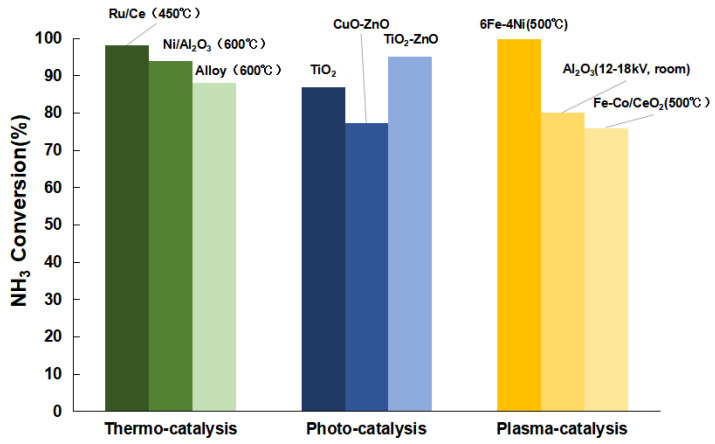
A comparison of various technical methods for ammonia decomposition. (As the evaluation of electrocatalytic NH_3_ decomposition for H_2_ production is mainly based on current density, no comparison is made here with other technical methods) [10,19,20,21,22,23,24,25].

To produce clean H_2_, various technologies have been developed, such as thermocatalytic NH_3_ decomposition, photocatalytic NH_3_ decomposition, plasma-catalytic NH_3_ decomposition, and electrocatalytic NH_3_ decomposition. Figure 2 shows the advantages and disadvantages of each method [10,11,13,14,15,16,17,18]. Understanding these methods is key to determining the current capabilities of the NH_3_-to-H_2_ conversion technologies. Figure 3 compares the performance of various technical methods for ammonia decomposition. The results were obtained under different conditions, enabling a thorough evaluation of the effectiveness of each approach [10,19,20,21,22,23,24,25]. Further discussion has been explored regarding the benefits and drawbacks of each approach below.

### 2.1. Thermocatalytic NH_3_ Decomposition

**Figure 4:**
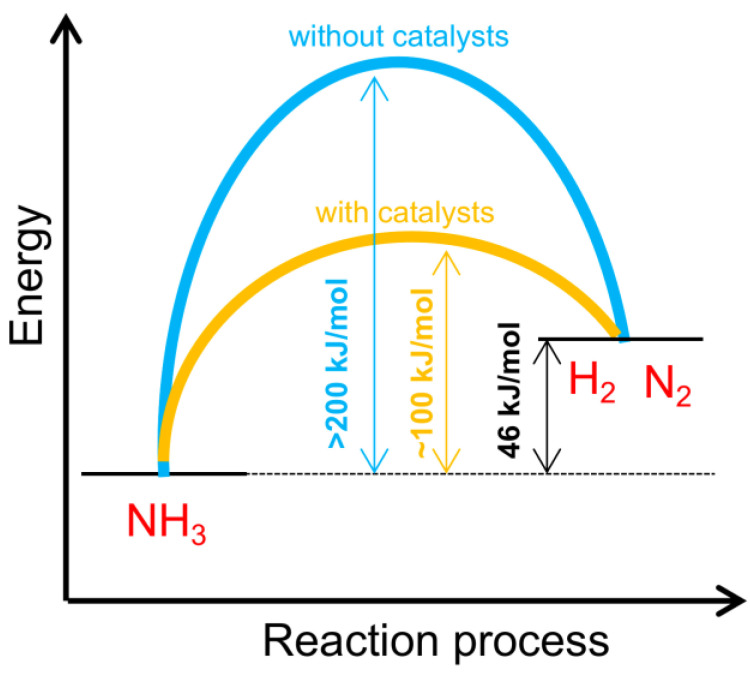
Energy profiles for ammonia decomposition without catalyst and with catalyst.

It should be noted that without a catalyst, the temperature needed for ammonia decomposition is quite high. This is due to the strong hydrogen bonds present within ammonia molecules, which demand a high level of energy to break apart. As a result, ammonia molecules break down into hydrogen and nitrogen [10,13,26,27]. Therefore, ammonia decomposition without a catalyst is not practical, as it necessitates high temperature and energy consumption as well as having a low reaction rate. Consequently, catalysts have been created to hasten the sluggish kinetics of NH_3_ decomposition and stimulate H_2_ production, as shown in Figure 4.

**Scheme 1:**
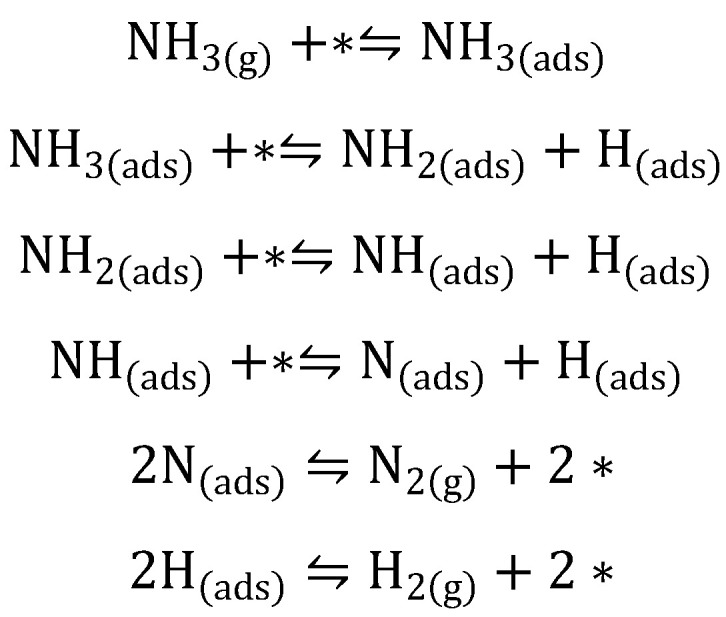
Elementary kinetic steps for the ammonia decomposition reaction; (*) refers to an unoccupied adsorption site on the catalyst [29].

The catalytic dissociation of ammonia can be explained as a process where ammonia molecules are absorbed into the active site of the catalyst, leading to a sequence of reactions involving dehydrogenation and recombinative desorption [7,9,28]. The reaction is generally accepted to occur in several steps, as shown in Scheme 1. Firstly, there is a molecular adsorption of NH_3_ that generates surface NH_3_* at the active sites of metal catalysts. Then, NH_3_* undergoes incremental dehydrogenation, resulting in the production of N* and H* atoms. Finally, N* and H* undergo recombinative desorption, leading to the release of N_2_ and H_2_ into the gas phase. These reactions culminate in the creation of di-nitrogen and di-hydrogen on the surface of the catalyst.

Regarding the ammonia synthesis reaction, dissociative nitrogen adsorption is typically viewed as the rate-determining step. Nevertheless, there’s a possibility that recombinative nitrogen desorption could also impact the rate of the decomposition reaction. This matter requires further investigation and research to determine its actual impact on the reaction kinetics. Lucentini et al. conducted a survey and analysis of various catalytic systems. The technological status and challenges of ammonia decomposition were first presented in their paper. Then, state-of-the-art catalytic systems and related reaction mechanisms were described in detail. Finally, the structured reactors for the decomposition reaction of ammonia were explored [10]. In summary, the nitrogen desorption is the dominant slow step, but the complexity of the reaction increases with the catalyst composition, active phase, and reaction conditions.

Regarding the catalysts, there has been a growing interest in using transition metal-based catalysts for NH_3_-to-H_2_ conversion. Although Ru-based catalysts have demonstrated excellent performance, they are less practical for large-scale applications due to their high cost [30,31,32]. Therefore, Fe and Ni-based catalysts have emerged as promising alternatives due to their superior performance, but efforts are still underway to further reduce costs, optimize activity, and increase their operational lifespan [33,34,35,36,37,38]. One approach to improving the activity of these catalyst systems is to use rare earth element-based heteroatoms, such as La and Ce, for doping. This results in improving the surface properties, increasing the number of active sites, and enhancing the catalytic activity of Fe and Ni-based catalysts [39,40,41,42,43]. Another approach is to create multi-metal alloy-based structures, which can further enhance the catalytic activity by providing better dispersion of the metal atoms, improving the stability of the catalyst, and increasing the resistance to poisoning [28,44,45,46,47,48,49].

The conventional process of H_2_ production through thermocatalytic NH_3_ decomposition is slow. It requires external energy input to heat the catalyst to at least 400 ℃ for a sufficient time, resulting in time delay and low energy efficiency. Therefore, a more suitable approach for the H_2_ production process is needed that does not require external energy input. One such approach involves exposing a pretreated catalyst to a mixture of NH_3_ and O_2_ gases at a specific ratio at room temperature. This approach is known as low-temperature ammonia oxidation, and it removes the need for the high-temperature decomposition of NH_3_. The reaction proceeds as follows:

$${NH}_{3}+1/4O_{2}\to H_{2}+1/2N_{2}+1/2H_{2}O\;(∆H=-75\;kJ/mol)$$

**Figure 5:**
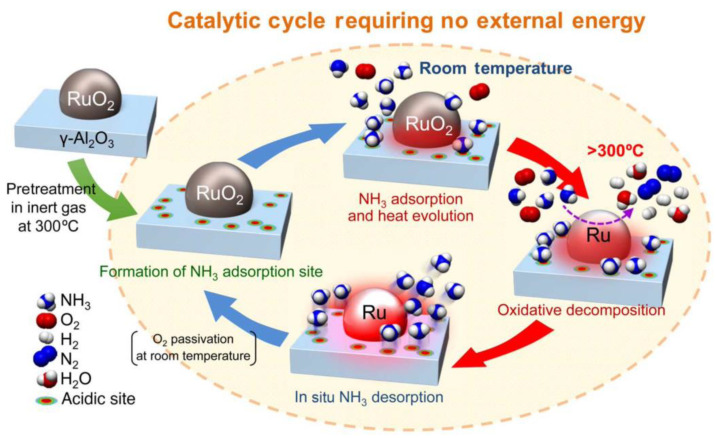
Schematic of the catalytic cycle developed for oxidative decomposition of ammonia (reproduced with permission from [50], Copyright Science, 2017).

Katsutoshi Nagaoka et al. studied hydrogen production using an acidic RuO_2_/γ-Al_2_O_3_ catalyst by exposing ammonia and O_2_ at room temperature, as shown in Figure 5 [50]. The exothermic adsorption of ammonia on the catalyst causes the catalyst bed to rapidly heat up to the auto-ignition temperature of catalytic ammonia, resulting in the oxidative decomposition of ammonia to generate hydrogen. A differential calorimeter and a volumetric gas sorption analyzer were used in the study to measure the heat released by the physisorption of multiple ammonia molecules and the chemisorption of ammonia onto both the RuO_2_ and acidic sites on the γ-Al_2_O_3_. The results showed a significant amount of heat evolved during both processes.

### 2.2. Non-Thermal Plasma-Catalytic NH_3_ Decomposition

Thermocatalytic decomposition of NH_3_ generally occurs at a temperature above 873 K, while traditional thermal reforming or decomposition of NH_3_ typically requires a high temperature of about 1300 K. Despite the presence of a catalyst, the reaction temperature is still relatively high, restricting the reaction’s useful applications. To start chemical reactions at lower temperatures, researchers have investigated the potential of alternate strategies involving electrical discharges or non-thermal plasmas [18,21,51]. Researchers have explored the potential of plasma-catalytic NH_3_ decomposition. The results show that non-thermal plasma can effectively transform NH_3_ into H_2_ with less influence from the gas temperature [52,53,54]. The results of studies conducted on plasma-catalytic NH_3_ decomposition suggest that it can be produced with lower energy consumption and higher efficiency [22,55,56,57].

Generally, the decomposition of ammonia induced through the direct application of plasma is restrained due to the following reasons: (1) Energy efficiency: Direct application of plasma does not now have any significant advantages in terms of energy use in contrast to catalytic decomposition [58,59,60]. This is because, beneath equilibrium conditions, ammonia can decompose at relatively low temperatures. (2) Plasma stabilization: When ammonia undergoes direct-discharge conditions, it decomposes to produce nitrogen and high concentrations of hydrogen [61]. This makes it challenging to stabilize the plasma, which similarly complicates the process of ammonia decomposition. However, one can still take advantage of the benefits of plasma, such as the speedy introduction of high-temperature reaction conditions. In the catalytic reaction system, plasma can function solely in the preliminary start-up stage segment till the catalyst bed is heated up to the reaction temperature [14]. In this case, hydrogen can be produced immediately at the beginning of the plasma system via the capability of a decomposition reaction. At the same time, the catalyst can be heated quickly via the warmth supplied via the plasma.

**Figure 6:**
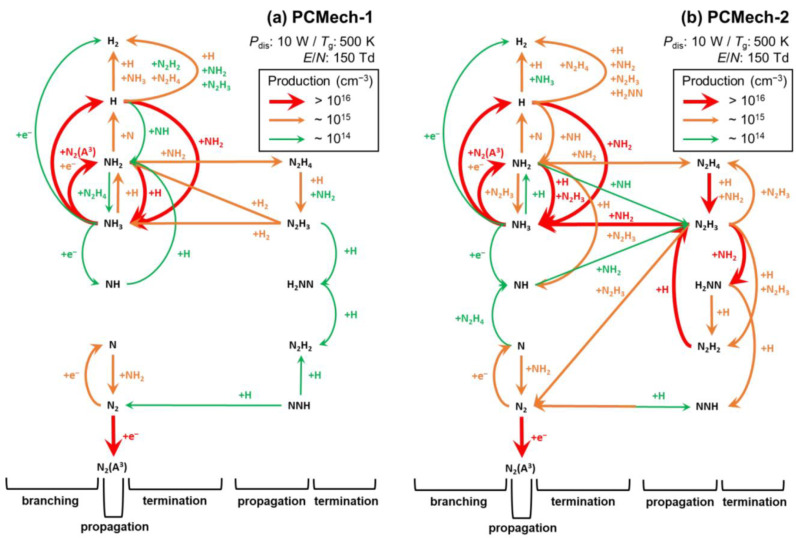
Schematic of the primary chain reactions for (**a**) PCMech^−1^ and (**b**) PCMech^−2^ for the NH_3_/N_2_ combination (1/99 mol%) with Tg = 500 K, E/N = 150 Td, and Pdis = 10 W. (reproduced with permission from [62], Copyright American Chemical Society, 2023).

The mechanism for the plasma-assisted conversion of NH_3_ can be divided into two parts. (1) Plasma mechanisms: These involve electron-induced chemistry, such as electron impact reactions, quenching of excited species, and ion reactions. The energetic electrons in the plasma interact with the NH_3_ molecules, leading to various chemical reactions. (2) Thermal mechanisms: These involve conventional thermal mechanisms for thermally induced chemistry. The heat generated by the plasma contributes to the overall conversion of NH_3_. Thermal energy helps break and form chemical bonds, facilitating the conversion process. Bang et al. conducted an experimental and numerical study of the plasma-assisted conversion of NH_3_ (Figure 6). They used a mixture of NH_3_ (1 mol%) diluted in N_2_ at atmospheric pressure. This allowed them to investigate the effects of plasma on NH_3_ conversion under specific conditions [62]. They observed that the conversion of NH_3_ took place even at room temperature during their study. They found a local peak in conversion at around 600 K. This finding suggests that the plasma-assisted cracking of NH_3_ has the potential to be cost-effective by reducing the temperature range required for the cracking reaction. Lowering the temperature window for the cracking reaction can lead to energy savings and improved efficiency in the conversion process.

### 2.3. Electrocatalytic NH_3_ Decomposition

The electrochemical process is also a promising alternative for onboard use, as hydrogen and nitrogen can be gained from the decomposition of ammonia at a moderate temperature [17,63,64]. Liquid ammonia has a theoretical electrolysis voltage of 0.077 V, significantly lower than water’s electrolysis voltage of 1.23 V [16,63]. When ammonia is electrolyzed, hydrogen molecules are produced at the cathode, and amide ions are released. Amide ions are oxidized at the anode to form nitrogen molecules. The electrolysis reactor must be designed as a highly closed electrolytic cell under stringent experimental conditions to prevent the oxidation and hydration of metal amides [63]. However, the current efficiency is only 85% at a high cell voltage of 2 V due to the inevitable reverse reaction in liquid ammonia. To make ammonia electrolysis practical, it is necessary to reduce the cell voltage as much as possible at a high current.

The electrocatalytic NH_3_ decomposition can occur in an aqueous electrolyte under certain pH conditions, either through the NH_3_ oxidation in an alkaline environment caused by OH^−^ ions after the adsorption of NH_3_ onto the electrode or through NH_4_^+^ oxidation by the oxidants such as hypochlorous acid in low pH environments [65,66,67]. However, electro-corrosion and sluggish kinetics in acidic electrolytes can lead to low efficiency in the electrochemical process. Alternative approaches to address this issue involve using alkaline electrolytes for electrocatalytic NH_3_ decomposition, which have been extensively studied and offer the potential to overcome undesirable problems related to acidic environments towards electrode materials. Ideally, in an NH_3_ decomposition system, NH_3_ is oxidized to N_2_ at the anode, while H_2_O is reduced to H_2_ at the cathode, producing the gaseous H_2_ and N_2_ from an ammonia solution. However, it is well known that the side reaction of OER may be a competing process with the Electrocatalytic NH_3_ decomposition at the anode.

Anode reaction:

$$2{NH}_{3}+6{OH}^{-}⟶N_{2}+6H_{2}O+6e^{-}\;E^{\theta }=-0.77\;V\;vs.\;SHE$$

Cathode reaction:

$$2H_{2}O+2e^{-}⟶H_{2}+2{OH}^{-}\;E^{\theta }=-0.829V\;\;vs.\;SHE$$

Overall reaction:

$$2{NH}_{3}⟶3H_{2}+N_{2}\;E^{\theta }=-0.059\;V$$

**Figure 7:**
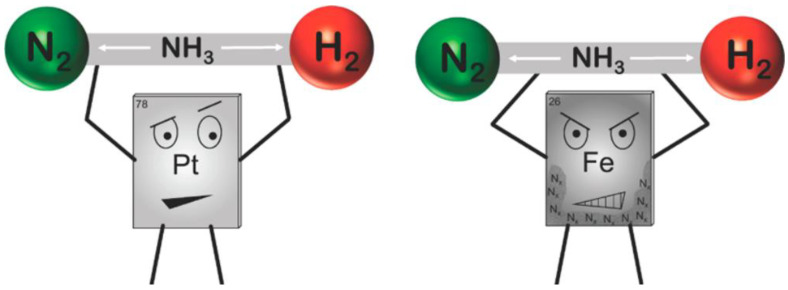
Pt and Fe catalysts decompose ammonia (reproduced with permission from [70], Copyright American Chemical Society, 2017).

The use of electrocatalytic NH_3_ decomposition to produce H_2_ has many benefits, including simplicity and cost-effectiveness. However, problems remain, such as slow kinetics and poor selectivity. To increase the electrocatalytic NH_3_ decomposition efficiency, various types of electrocatalysts have been studied, but their performance still cannot satisfy the requirements of industrial applicability. Therefore, it is important to develop new high-efficiency ammonia electrolysis electrodes for hydrogen production, as shown in Figure 7. Although platinum and other precious metal-based catalysts are effective for the process of electrocatalytic NH_3_ decomposition (alkaline water electrolysis), their high cost and limited availability are significant drawbacks [16,17,68,69,70]. Recently, catalysts based on transition metals have shown promise for electrocatalytic NH_3_ decomposition through various structural and morphological engineering techniques, including alloyed/core-shell formation, shape control, heteroatom doping, and self-supporting materials [40,66,71,72,73]. However, the current density of oxidation and selectivity achieved by these catalysts is still lower than what is required for practical application. Therefore, further research and development in this area is necessary.

### 2.4. Photocatalytic NH_3_ Decomposition

**Figure 8:**
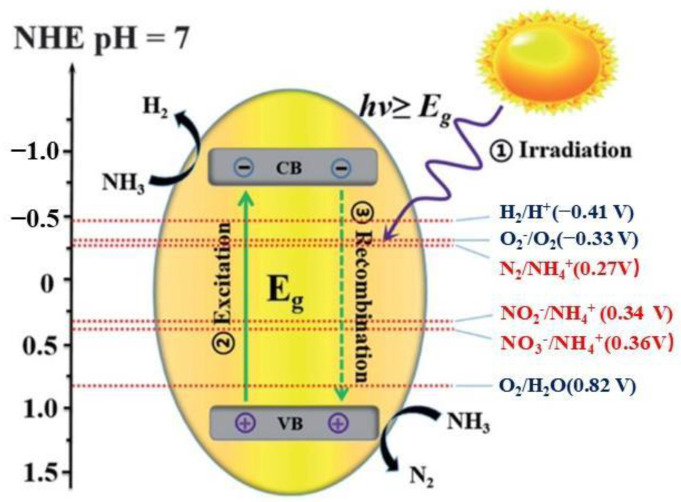
Illustration of the process of photocatalytic ammonia decomposition (reproduced with permission from [15], Copyright Royal Soc Chemistry, 2020).

The photocatalytic method of splitting NH_3_ into N_2_ and H_2_ is also a promising approach, as it can be carried out using recyclable catalysts at room temperature and can easily control light exposure with a switch. Using sunlight to decompose ammonia through photocatalysis is an artificial photosynthetic reaction that occurs in alkaline conditions [15]. As shown in Figure 8, when the energy of the irradiating light exceeds the band gap of the photocatalyst it creates electron-hole pairs. The resulting holes act as strong oxidants, while the electrons in the conduction band can reduce O_2_ to form hydroxyl radicals. To achieve the photocatalytic degradation of ammonia, the electrons and holes generated on the surface of a semiconductor must have appropriate reduction and oxidation abilities to interact with the adsorbed species on the catalyst surface and produce free radicals or other products.

$$Photocatalyst+hv⟶e^{-}+h^{+}$$

$$2{NH}_{3}+6h^{+}⟶N_{2}+6H^{+}$$

$$2H^{+}+2e^{-}⟶H_{2}$$

**Figure 9:**
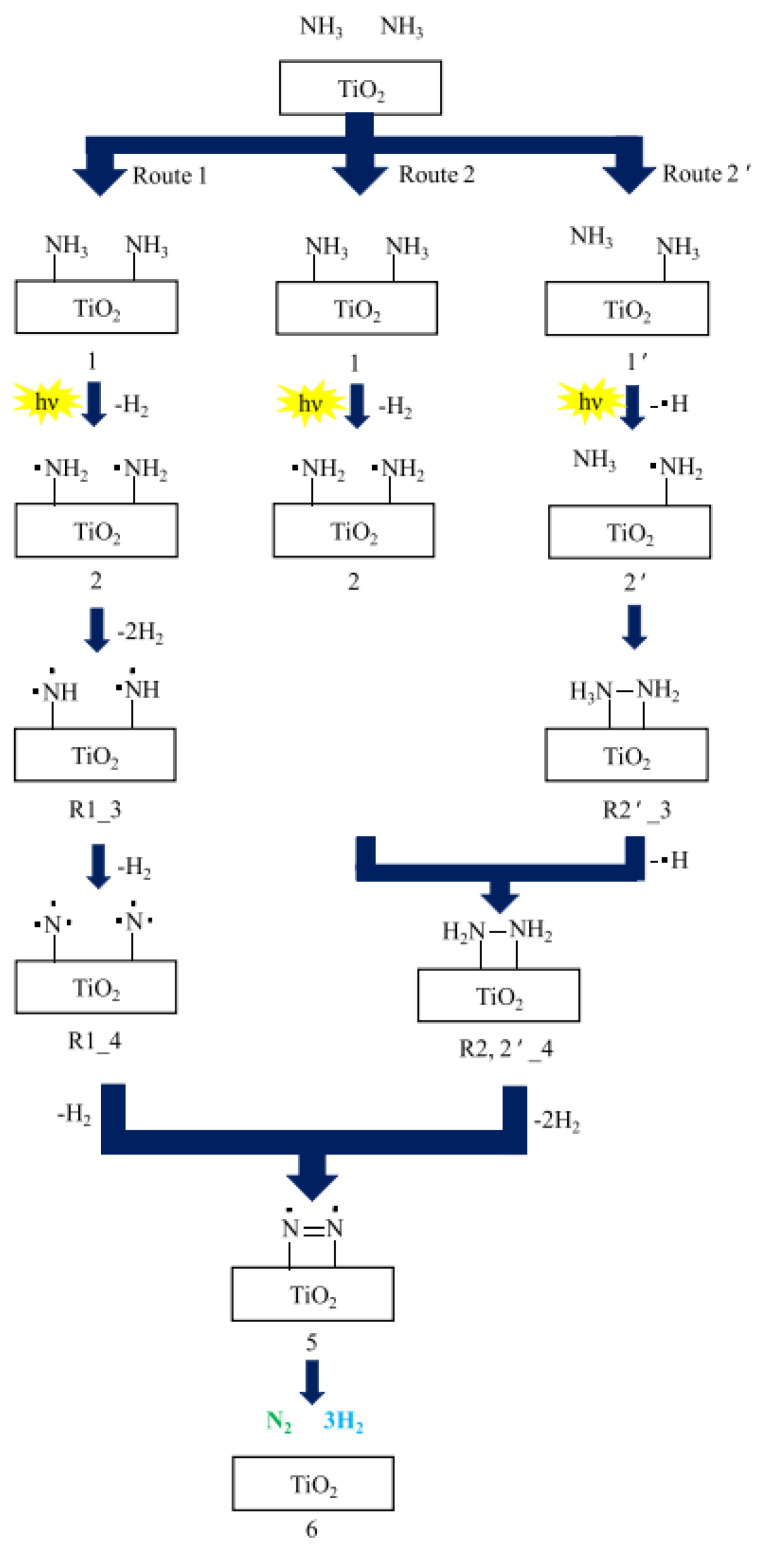
Reaction mechanism of NH_3_ decomposition to N_2_ and H_2_ over TiO_2_ photocatalyst (reproduced with permission from [83], Copyright Elsevier, 2017).

So far, only a limited number of photocatalysts have been found to be effective in the decomposition of an aqueous ammonia solution. These include common photocatalysts, such as TiO_2_, ZnO, ZnS, Mo_2_N, and graphene, as well as their hybrid materials that are loaded with metals [15,74,75,76,77,78,79,80,81,82,83]. Utsunomiya et al. studied the photocatalytic activity of TiO_2_ loaded with different metals on the decomposition of ammonia and explored the NH_3_ decomposition mechanism by proposing three reaction pathways [83]. As shown in Figure 9, route 1 involves the formation of NH radicals by removing one hydrogen atom from each of the two NH_2_ radicals, while route 2 involves the coupling of adjacent NH_2_ radicals to form NH_2_-NH_2_. Route 2′ involves the formation of NH_2_-NH_2_ through the formation of H_2_N-NH_3_. The activation energies for routes 1 and 2 calculated by density functional theory (DFT) are 236 kcal/mol and 74.8 kcal/mol, respectively. Of these pathways, route 2 is considered energetically more favorable than route 1. Possible reaction pathways for the formation of N_2_ and H_2_ through NH_2_-NH_2_ coupling were further divided into two routes: route 2, which involves the coupling of NH_2_ radicals to form H_2_N-NH_2_, and route 2′, which involves the interaction of NH_2_ with one NH_3_ molecule in the gas phase. The activation energies of the two routes were estimated to be 74.4 kcal/mol and 59.2 kcal/mol, respectively. Therefore, it is plausible that NH_3_ decomposition occurs via the formation of NH_2_-NH_2_ through routes 2 and 2′. Razak et al. reported the linear generation of H_2_ from NH_3_ over Pd/TiO_2_ catalyst. They suggest that the production of H_2_ from NH_3_ was initiated by the interaction of N atoms on the Pd surface. This interaction led to the dissociation of N-H bonds under the catalysis of photogenerated electrons [84].

**Figure 10:**
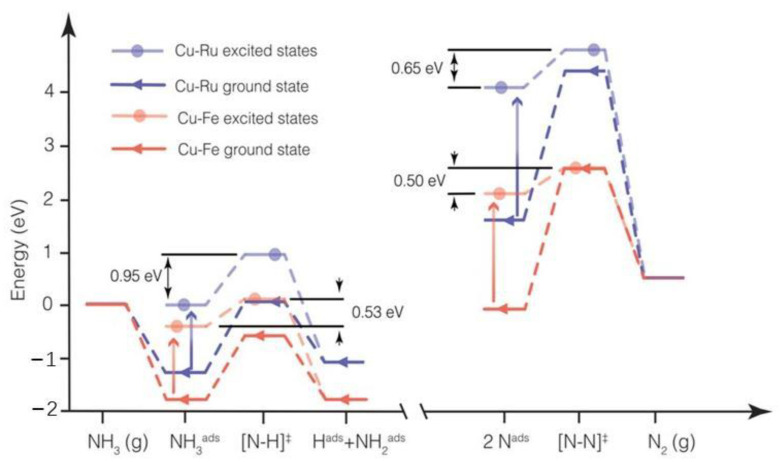
Schematic comparison of the energy diagram for photocatalysis (excited states) and thermocatalysis (ground state) on Cu-Fe- and Cu-Ru-ARs. Red arrows refer to electronic excitation on Fe-N, and blue to Ru-N. Only the two possible RDSs are included for simplicity; migrations of nitrogen species are not considered for the same reason (reproduced with permission from [85], Copyright Science, 2022).

Recently, Yuan et al. have discovered that Cu-Fe-AR, which is not an effective thermocatalyst, can function as an excellent photocatalyst for NH_3_ decomposition when exposed to short-pulse laser illumination [85]. The formation of adsorbate–metal excited states by hot carriers leads to the lowering of activation barriers, active site cleaning, and product desorption, resulting in enhanced reactivity and stability of Cu-Fe-AR compared to traditional thermocatalysts. Under continuous-wave LED illumination, Cu-Fe-AR manifests a high efficiency comparable to Cu-Ru-AR. Although Cu-Ru-AR exhibits slightly greater reactivity due to photothermal heating, the study demonstrates that photocatalysis can be efficiently performed with inexpensive LED photon sources, as shown in Figure 10. The results suggested that abundantly available metals might serve as productive and cost-effective catalyst substrates for plasmonic antenna-reactor photocatalysis.

## 3. Catalysts for NH_3_ Decomposition

The choice of catalyst is critical for the efficient decomposition of ammonia to produce hydrogen at low temperatures, and different technologies require different catalysts. For example, the Ru group of catalysts is commonly used in thermocatalytic NH_3_ decomposition, while the Pt group is suitable for both electrocatalytic and photocatalytic NH_3_ decomposition. In addition to co-catalysts, such as Ru and Pt, photocatalysts are also required in photocatalytic NH_3_ decomposition [9,26,72,73]. In non-thermal plasma-catalytic NH_3_ decomposition, Fe-based catalysts have been the most studied [48,51,54]. Furthermore, for the industrialization of hydrogen production from ammonia decomposition, catalyst support is vital as it has a synergistic effect on the catalyst’s performance. Therefore, this review will focus on precious metal catalysts, non-precious metal catalysts, multi-alloy catalysts, and other aspects, emphasizing the research emphasis on various catalysts in different NH_3_ decomposition techniques. By understanding the unique roles catalysts play in different technologies, researchers can optimize performance and improve the overall efficiency of the ammonia decomposition process.

### 3.1. Precious Metal Catalysts

**Figure 11:**
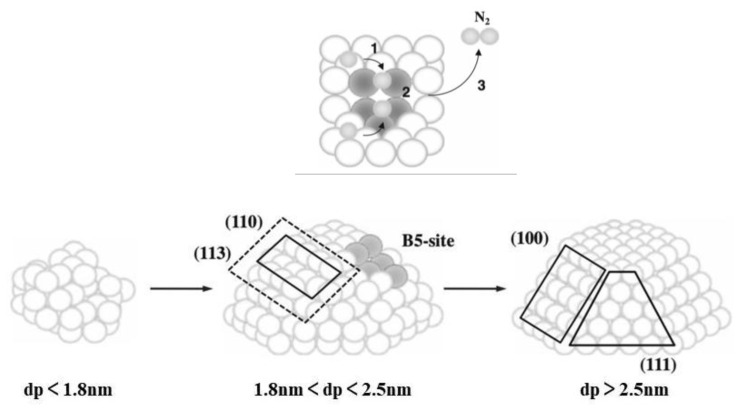
Scheme of associative adsorption of the two N atoms to form an N_2_ molecule in a B5-type site: (1) N atoms diffusion to the B5-type site, (2) association of N atoms to form an N_2_ molecule, and (3) desorption of the N_2_ molecule (‘three stages of the growth of an Ru particle’ reproduced with permission from [91], Copyright Springer, 2009).

Precious metals are a significant type of catalyst material, with Ru-based materials being particularly noteworthy for their exceptional performance in NH_3_ decomposition [7,9,10,31]. As a result, they have been extensively studied. The catalysis of NH_3_ decomposition on Ru-based catalysts is primarily attributed to B5 active sites [31,86,87,88]. The B5-type site comprises a configuration of three Ru atoms in one layer and two additional Ru atoms in the layer directly above it at a monoatomic step on a Ru(0001) terrace [89], as shown in Figure 11. Thus, the number of B5 active sites is frequently linked to the size and shape of Ru nanoparticles on the catalyst surface [90,91].

**Figure 12:**
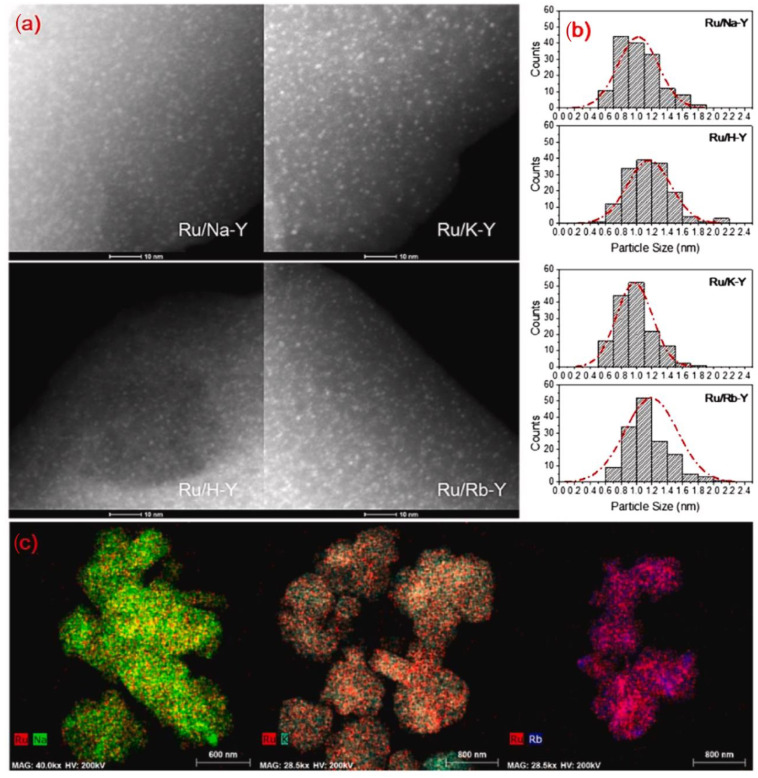
(**a**) HAADF-STEM images, (**b**) particle size distribution, and (**c**) EDS elemental mapping images of Ru/M-Y catalysts (M = H, Na, K, and Rb) (reproduced with permission from [93], Copyright Elsevier, 2021).

Kim et al. investigated the influence of the crystalline phase of alumina on the decomposition of NH_3_ over Ru catalysts supported on alumina [87]. The results show that the dispersion and morphology of Ru are key factors for H_2_ production from NH_3_ decomposition over Ru/Al_2_O_3_ catalysts and can be regulated by the support and calcination temperature. Among the Ru/Al_2_O_3_ catalysts calcined at various temperatures and reduced at 573 K, Ru/γ-Al_2_O_3_ with Ru particle sizes of 7~8 nm displayed the highest rate of ammonia decomposition. Feng et al. have demonstrated that Yttrium oxide (Y_2_O_3_) can effectively disperse and stabilize Ru nanoparticles as a functional support for catalysts [92]. Highly dispersed Ru nanoparticles on Y_2_O_3_ (Ru/Y_2_O_3_) were prepared, and the catalytic activity of ammonia decomposition was evaluated. The results showed that at a relatively high weight gas hourly space velocity of 30,000 mLg^−1^h^−1^, the 5 wt.% Ru/Y_2_O_3_ catalyst exhibited excellent catalytic activity, achieving near-complete NH_3_ conversion at 475 °C, superior to many Ru catalysts supported by typical oxides. Cha et al. reported the successful loading of highly monodisperse Ru particles onto alkali-exchanged zeolite Y support using an ion-exchange method coupled with a vacuum calcination treatment [93]. As shown in Figure 12, the resulting Ru/M-Y catalysts exhibit an average particle size of approximately 1 nm and contain both nanometer- and sub-nanometer-sized Ru particles. Hu et al. reported the successful synthesis of stable ruthenium (Ru) clusters with a size of about 1.5 nm by reduction of single Ru atoms under an ammonia atmosphere at 550 °C [94]. These Ru clusters are uniformly dispersed on the surface of the ceria. The obtained supported ruthenium cluster catalysts exhibit exceptional activity for ammonia decomposition with an extremely high hydrogen production rate. Ru-based materials have potential applications in Photocatalytic NH_3_ decomposition and Plasma-catalytic NH_3_ decomposition. Iwase et al. have reported that a Ru-loaded ZnS photocatalyst demonstrated remarkable activity in decomposing an aqueous ammonia solution into H_2_ and N_2_ with a stoichiometric amount under simulated solar radiation [79]. In this reaction, the Ru co-catalyst was crucial in promoting ammonia oxidation to N_2_.

**Figure 13:**
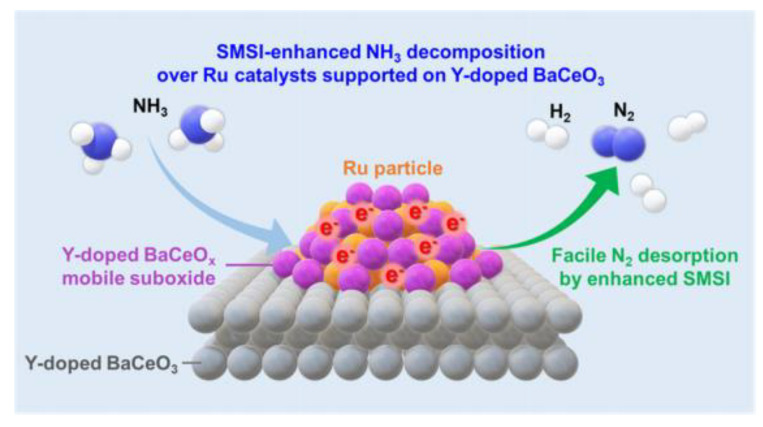
Schematic diagram of NH_3_ decomposition of Ru/Y-dosed BaCeO_3_ catalyst (reproduced with permission from [95], Copyright American Chemical Society, 2022).

On the other hand, the support used also strongly affects the catalytic activity of Ru-based materials. Firstly, a support with a high specific surface area can enhance the dispersal of Ru nanoparticles, leading to improved NH_3_ adsorption capacity [92,93,94]. Secondly, the characteristics of the support itself can have a synergistic effect with Ru nanoparticles, thereby enhancing catalytic activity [23,95,96,97,98,99]. For example, Jeon et al. synthesized a Y-doped BaCeO_3_ perovskite and constructed a strong metal support interaction (SMSI) interface with Ru particles, as shown in Figure 13 [95]. The catalyst achieved 100% NH_3_ decomposition efficiency at 500 °C and had a higher H_2_ generation rate than most Ru-based catalysts. The improvement in catalytic performance can be attributed to the promotion of N_2_ desorption by the SMSI interface. Yamazaki et al. studied Ru catalysts supported on CeO_2_−PrO_x_ composites (Ru/CP) prepared through coprecipitation [99]. The study found that the activity of the Ru/CP catalyst is higher than that of the Ru/CeO_2_ and Ru/PrO_x_ catalysts, and the Pr content (=Pr/(Ce + Pr)) reached the target value of 33–67%. It was also revealed that increasing the Pr content in the catalysts enhanced the Ru dispersion but suppressed Ru metalation due to a strong “metal–support interaction”.

**Figure 14:**
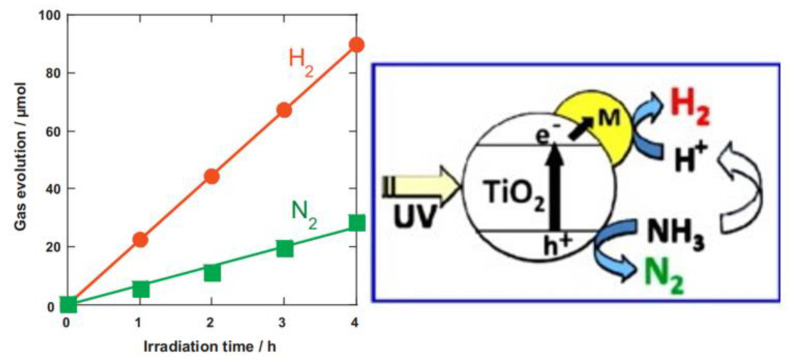
Schematic diagram of Pt/TiO_2_ photocatalytic NH_3_ decomposition performance and reaction mechanism (reproduced with permission from [76], Copyright Elsevier, 2012).

Apart from Ru-based materials, Pt-based materials are also extensively studied in the field of NH_3_ decomposition. While Ru-based materials are mainly used for thermocatalytic NH_3_ decomposition, Pt-based materials are typically employed for photocatalytic and electrocatalytic NH_3_ decomposition [16,17,68,69,75,76,100]. In a study by Kominami et al., the photocatalytic NH_3_ decomposition performance of metal-supported TiO_2_ catalysts was investigated [76]. Pt/TiO_2_ exhibited the best performance, with the amount of H_2_ and N_2_ reaching 90 and 28 umol, respectively, after 4 h of light irradiation, as shown in Figure 14. The H_2_/N_2_ ratio was 3.2, consistent with the stoichiometric ratio of NH_3_ decomposition. Dong et al. successfully electrolyzed liquid ammonia with metal amide as the supporting electrolyte using Pt electrodes [16]. In a subsequent study, they replaced the Pt electrode with a Pt/Rh/Ir alloy electrode, which tripled the current density [69].

### 3.2. Non-Precious Metal Catalysts

Although precious metal materials have excellent NH_3_ decomposition ability, their high cost as precious metals limits their potential for large-scale applications. Therefore, researchers are exploring the development of non-precious metal catalysts. Currently, non-precious metals such as Ni, Co, and Fe have demonstrated good NH_3_ decomposition activity and are being investigated as potential alternatives [6,9,10,101].

**Figure 15:**
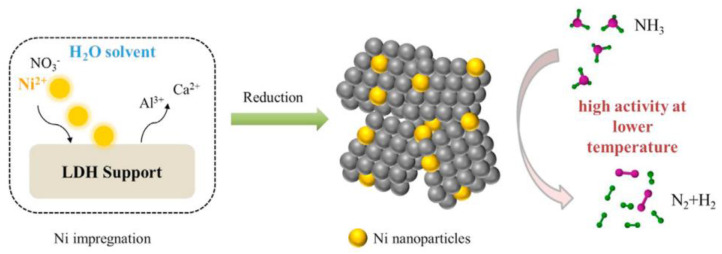
Schematic illustration of the synthesis of Ni nanoparticles derived from the thermal reduction of Ni-containing Ca-Al LDH (reproduced with permission from [104], Copyright Elsevier, 2021).

Ni-based catalysts have received significant attention among numerous non-precious metal catalysts due to their lower cost and significant activity [7,102]. The activity of Ni-based catalysts is primarily influenced by the size of Ni nanoparticles present on the catalyst surface [103,104,105]. Deng et al. obtained Ni nanoparticles with varying particle sizes through direct thermal reduction of nickel-containing Ca-Al layered double hydroxides (LDH) and tested them for NH_3_ decomposition, as shown in Figure 15 [104]. Among these catalysts, the Ni/CaAlO_x_ catalyst with Ni(NO_3_)_2_ as the precursor exhibited the best NH_3_ decomposition performance, with an NH_3_ conversion rate of 99% at 550 °C. This is because the average particle size of the Ni nanoparticles, reduced with Ni (NO_3_)_2_ as the precursor, is the smallest (4.7 nm), allowing for better dispersion on the surface of CaAlO_x_ and, thus, more effective adsorption of NH_3_.

Besides the size of Ni nanoparticles, the support used in Ni-based catalysts also plays a crucial role in their catalytic activity. Currently, metal oxides such as CeO_2_ [43,106,107,108,109], Y_2_O_3_ [110,111], Al_2_O_3_ [112,113,114], and La_2_O_3_ [115] are commonly used as supports for Ni-based catalysts used in NH_3_ decomposition. The high activity of these metal oxide-supported Ni-based catalysts is typically due to the high dispersity of Ni nanoparticles, the strong interaction between the support and Ni nanoparticles, and the presence of oxygen vacancies on the support surface. Li et al. successfully used a Ni single-atom supported CeO_2_ (SA Ni/CeO_2_) catalyst for NH_3_ decomposition into H_2_ under solar heating conditions [109]. The experimental results showed that at 300 °C, the rate of NH_3_ decomposition to H_2_ reached 3.544 mmol g^−1^ min^−1^, which was superior to all non-precious metal catalysts and most precious metal catalysts. Based on this, they combined their self-made solar heating system, and the H_2_ generation rate of the catalyst under 1 sun reached 1.58 mmol g^−1^ min^−1^, which was 100 times higher than the previous record. Do et al. synthesized a series of Ni/AlCeO_x_ composites using the cation-anion double hydrolysis method and used them as catalysts for NH_3_ decomposition to H_2_ production [114]. The experimental results showed that the catalyst exhibited excellent catalytic performance and could completely decompose NH_3_ at around 550 °C. The improved catalytic performance can be attributed to the highly dispersed and reducible Ni atoms, the increased amount of surface oxygen vacancies, and the significantly enhanced NH_3_ adsorption affinity.

**Figure 16:**
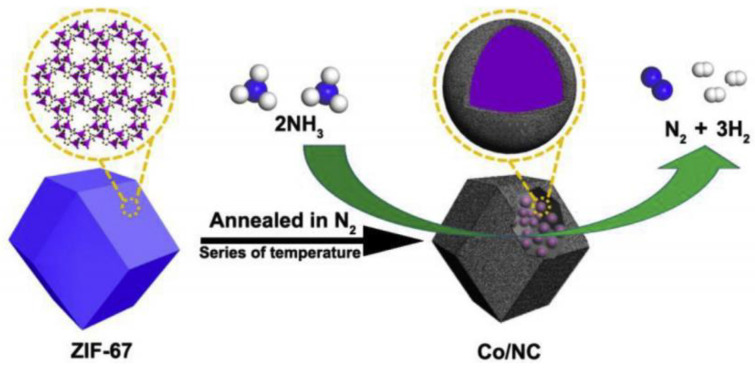
Schematic illustration of the synthesis of Co/NC-X (reproduced with permission from [125], Copyright Elsevier, 2022).

Co-based materials have also been extensively studied as catalysts for NH_3_ decomposition [116,117,118]. Many properties of Co-based catalysts are similar to those of Ni-based catalysts, and their catalytic activity is also influenced by the size of Co nanoparticles and the support structure [119,120,121,122,123,124,125]. Lei et al. successfully prepared small Co nanoparticles dispersed on N-doped carbon carriers (Co/NC-X) by pyrolyzing ZIF-67 in an N_2_ atmosphere at different temperatures (X = 500, 600, 700, 800 °C). They used them as catalysts for NH_3_ decomposition to H_2_, as shown in Figure 16 [125]. The Co NPs were evenly distributed and highly dispersed on NC, resulting in high catalytic activity. Among these catalysts, the NH_3_ conversion rate of Co/NC-600 at 500 °C was 80%, and the H_2_ production rate was 26.8 mmol g^−1^ min^−1^. Yu et al. investigated the effect of BaNH, CaNH, and Mg_2_N_3_ on the catalytic activity of Co in the NH_3_ decomposition reaction [124]. In the reaction temperature range of 300–550 °C, the formation rate of H_2_ was in the order of Co-BaNH > Co-CaNH > Co-Mg_2_N_3_. Notably, the H_2_ generation rate of Co-BaNH at 500 °C reached 20 mmol g^−1^ min^−1^, comparable to the active Ru/Al_2_O_3_. In-depth studies revealed that a [Co-N-Ba]-like intermediate species was formed at the interface of Co metal and BaNH, which led to the higher catalytic activity of Co-BaNH.

**Figure 17:**
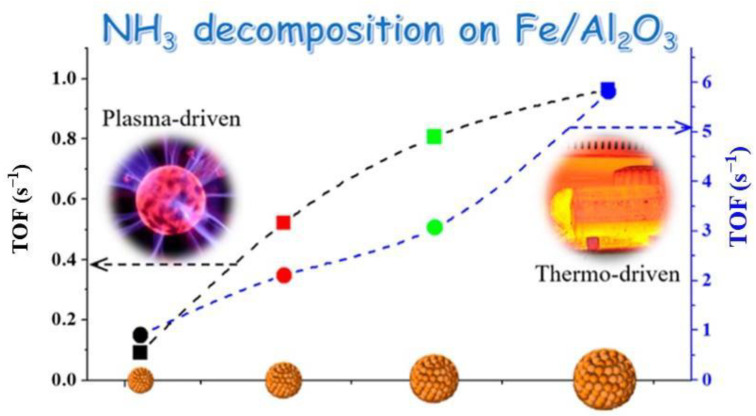
Schematic diagram of NH_3_ decomposition ability of Fe/Al_2_O_3_ catalysts driven by plasma and thermo as the size of Fe_3_O_4_ nanoparticles increases (reproduced with permission from [57], Copyright American Chemical Society, 2022).

The NH_3_ decomposition activity of Fe-based materials is generally lower than that of Ni-based materials and Co-based materials due to the higher bond energy of Fe-N compared to Ni-N and Co-N [9,101]. However, despite this, researchers still have great interest in Fe-based materials due to their low price [36,37,38,52,55,57,126,127]. Lu et al. found a hidden active phase in Fe-based catalysts for NH_3_ decomposition, with the highest dehydrogenation rate corresponding to an evanescent Fe/Fe4N mixing phase [38]. However, the deposition of excess nitrogen atoms on the Fe-based catalyst can cause deactivation and a decrease in catalytic efficiency. Chen et al. observed a “particle size effect” in the activity of Fe_3_O_4_/Al_2_O_3_ catalyst in NH_3_ decomposition, with TOFs increasing as the size of Fe_3_O_4_ nanoparticles increased [57]. As shown in Figure 17, this effect was also observed in plasma-catalyzed NH_3_ decomposition.

### 3.3. Multi-Metallic Catalysts

To overcome the limitations of single noble metal catalysts and single non-noble metal catalysts in the large-scale application of NH_3_ decomposition, researchers are exploring the use of multi-metallic catalysts. These catalysts offer a balance between cost and performance, with low cost, good catalytic performance, and high stability. Additionally, the composition and morphology of different metals in the catalysts can be regulated, providing more possibilities for optimization.

**Figure 18:**
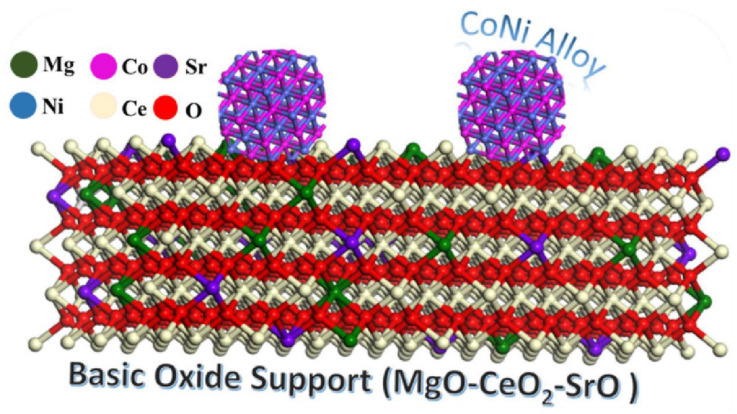
Schematic illustration for the decorated CoNi alloy on the oxide support of MgO–CeO_2_–SrO (reproduced with permission from [49], Copyright Royal Society of Chemistry, 2022).

At present, bimetallic catalysts are the mainstream of multi-metallic catalysts [28]. Common bimetallic catalysts include Ru-Ni [128,129,130], Ni-Co [42,46,49,131,132], Ni-Fe [47,48,133], etc. Hansgen et al. developed a computational framework that utilizes nitrogen binding energies to identify potential monolayer bimetallic catalysts [134]. Through this framework, they were able to predict that Ni-Pt-Pt(111) would be an effective bimetallic catalyst for NH_3_ decomposition, even exceeding the activity of Ru. Their calculations proved to be successful in identifying this high-performing catalyst. Tabassum et al. synthesized CoNi alloy nanoparticles that were well dispersed on mixed oxide support of MgO-CeO_2_-SrO, with potassium promotion, resulting in the K–CoNi_alloy_–MgO–CeO_2_–SrO catalyst, as shown in Figure 18 [49]. This catalyst exhibited high efficiency in converting NH_3_, with a conversion rate of 97.7% under reaction conditions of 450 °C and 6000 mLh^−1^gcat^−1^. The study suggests that the active sites located on the metal/oxide interface promote the recombination of adsorbed N atoms, leading to N_2_ desorption and a significant reduction in activation energy barriers. Shiraishi et al. developed a bimetallic alloy nanoparticle-supported TiO_2_ photocatalyst (Pt_0.9_Au_0.1_/TiO_2_) with 90 mol% Pt and 10 mol% Au [20]. This photocatalyst demonstrated effective decomposition of NH_3_ into H_2_ and N_2_, with significantly higher catalytic activity than Pt/TiO_2_. The enhanced performance can be ascribed to the presence of Au in the alloy, which reduces the Schottky barrier height at the metal/TiO_2_ interface, leading to a more efficient transfer of conduction band electrons on TiO_2_ to the metal particles. Yi et al. conducted a study where they prepared various bimetallic catalysts (Fe-Co, Mo-Co, Fe-Ni, and Mo-Ni) and utilized them for plasma-catalyzed NH_3_ decomposition [22]. The results revealed that the Fe-Ni catalyst displayed the highest catalytic activity compared to the other catalysts. Further analysis indicated that the Fe-Ni catalyst had the highest NH_3_ adsorption capacity, likely the primary reason for its superior catalytic activity. Jiang et al. investigated various morphologies of NiCo_2_N compounds. They specifically focused on the structure of nanoneedles grown on 3D nickel foam. It was found that this particular structure exhibited several advantages, including a significantly increased surface area, more exposed active sites, enhanced charge transfer, and improved gas diffusion. Furthermore, the NiCo_2_N composite demonstrated the most optimal catalytic activity in both the hydrogen evolution reaction (HER) and ammonia electrolysis [135].

**Figure 19:**
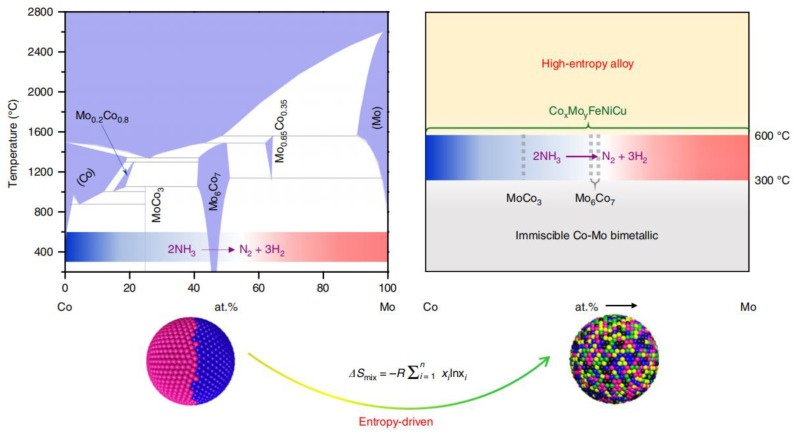
HEA catalysts breaking the miscibility limitation of conventional binary alloys (reproduced with permission from [44], Copyright Nature, 2019).

Developing multi-metal catalysts containing three or more metal elements is a more challenging task, but it also holds greater potential. Xie et al. successfully utilized a novel high-entropy alloy (HEA) CoMoFeNiCu nanoparticles for highly efficient NH_3_ decomposition [44]. As illustrated in Figure 19, they overcame the miscibility limit of bimetallic CoMo alloys by stably tuning the Co/Mo elemental ratio in CoMoFeNiCu HEA nanoparticles. The HEA catalyst has a lower cost than Ru-based catalysts and exhibits better catalytic performance than Co-Mo catalysts. At a reaction temperature of 500 °C, the optimal conversion efficiency of NH_3_ using this catalyst can reach 100%. Furthermore, the alloy composition and surface adsorption properties of the HEA catalyst can be well-tuned, demonstrating its immense potential for practical applications.

### 3.4. Other Catalysts

**Figure 20:**
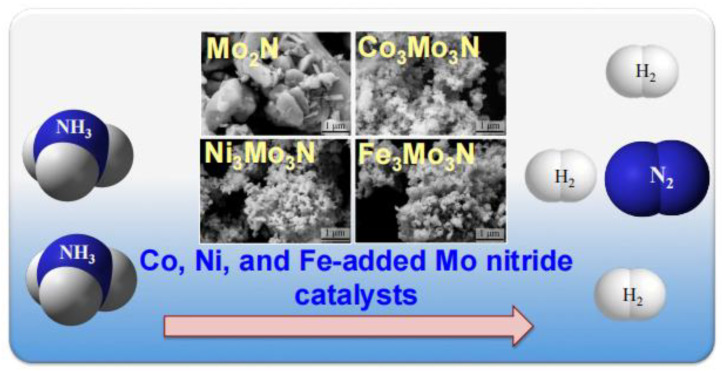
Schematic diagram of Mo_2_N catalysts with the addition of Co, Ni, and Fe for NH_3_ decomposition (reproduced with permission from [141], Copyright Royal Society of Chemistry, 2016).

In addition to metal nanoparticles, some metal nitrides can also be used as NH_3_ decomposition catalysts, such as Mo_2_N [136,137,138], MnN [139], Co_3_Mo_3_N [140,141,142], etc. Huo et al. designed Mo_2_N nanocrystals anchored on two-dimensional (2D) mesoporous silica/reduced graphene oxide (rGO) hybrid nanosheets (Mo_2_N/SBA-15/rGO) as an NH_3_ decomposition catalyst [136]. Benefiting from the highly dispersed Mo_2_N nanocrystals and modest Mo-N band strength, the catalyst exhibits excellent catalytic performance with an ammonia decomposition rate as high as 30.58 mmol g^−1^ min^−1^. Srifa et al. synthesized Mo_2_N-based catalysts with Fe, Ni, and Co additives, as shown in Figure 20 [141]. The NH_3_ decomposition activities of Co_3_Mo_3_N, Ni_3_Mo_3_N, and Fe_3_Mo_3_N catalysts were higher than Mo_2_N catalysts. Among them, Co_3_Mo_3_N has the best catalytic performance, and the NH_3_ conversion efficiency reaches 94% at 550 °C. The increase in catalytic activity can be attributed to adding Co, Ni, and Fe to increase the particle size and specific surface area of the catalyst and promote the recombination and desorption of N atoms.

Researchers have also developed some NH_3_ decomposition catalysts of sulfides and carbides, but the performance is average. Kraupner et al. synthesized mesoporous Fe_3_C with high crystallinity [143]. The material has transmission pores with a diameter of 20 nm and a high specific surface area (about 415 m^2^ g^−1^). At 700 °C, the conversion rate of NH_3_ is over 95%. Krishnan et al. successfully synthesized MoS_2_-supported acid, and alkaline functionalized (Al, Ti, and Zr)-Laponite catalysts [144]. The results show that the presence of heteroatoms (Al, Ti, and Zr) in the Laponite framework plays a key role in improving the reducibility, acid-base functional groups, and textural properties of MoS_2_. Among them, the MoS_2_/Zr-Laponite catalyst has the best NH_3_ decomposition performance, and its NH_3_ conversion rate at 600 °C degrees is 94%.

## 4. Conclusions

Ammonia decomposition is a highly efficient, environmentally friendly, and sustainable technology for hydrogen production that has attracted significant attention. This article explores the technical path and catalysts used for hydrogen production through ammonia decomposition. The article discusses various technologies and their catalytic mechanisms, highlighting their advantages and disadvantages and their potential for industrialization. The review also covers recent progress in catalyst research for ammonia decomposition, emphasizing the importance of finding non-precious metals to reduce costs and improve efficiency. Overall, this article provides valuable insights into the current state of NH_3_ decomposition for H_2_ production and the potential for future advancements in this field.

## Data Availability

All necessary data of this review has been included in the paper and no further data is needed.

## References

[1] Awad O.I., Zhou B., Harrath K., Kadirgama K. (2022). Characteristics of NH_3_/H_2_ blend as carbon-free fuels: A review. Int. J. Hydrogen Energy.

[2] Wan Z., Tao Y., Shao J., Zhang Y., You H. (2021). Ammonia as an effective hydrogen carrier and a clean fuel for solid oxide fuel cells. Energy Convers. Manag..

[3] Klerke A., Christensen C.H., Nørskov J.K., Vegge T. (2008). Ammonia for hydrogen storage: Challenges and opportunities. J. Mater. Chem..

[4] Chang F., Gao W., Guo J., Chen P. (2021). Emerging Materials and Methods toward Ammonia-Based Energy Storage and Conversion. Adv. Mater..

[5] Makhloufi C., Kezibri N. (2021). Large-scale decomposition of green ammonia for pure hydrogen production. Int. J. Hydrogen Energy.

[6] Sun S., Jiang Q., Zhao D., Cao T., Sha H., Zhang C., Song H., Da Z. (2022). Ammonia as hydrogen carrier: Advances in ammonia decomposition catalysts for promising hydrogen production. Renew. Sustain. Energy Rev..

[7] Le T.A., Do Q.C., Kim Y., Kim T.-W., Chae H.-J. (2021). A review on the recent developments of ruthenium and nickel catalysts for CO_x_-free H_2_ generation by ammonia decomposition. Korean J. Chem. Eng..

[8] Lim D., Kim A., Cheon S., Byun M., Lim H. (2021). Life cycle techno-economic and carbon footprint analysis of H_2_ production via NH_3_ decomposition: A Case study for the Republic of Korea. Energy Convers. Manag..

[9] Mukherjee S., Devaguptapu S.V., Sviripa A., Lund C.R.F., Wu G. (2018). Low-temperature ammonia decomposition catalysts for hydrogen generation. Appl. Catal. B Environ..

[10] Lucentini I., Garcia X., Vendrell X., Llorca J. (2021). Review of the Decomposition of Ammonia to Generate Hydrogen. Ind. Eng. Chem. Res..

[11] Tran D.T., Nguyen T.H., Jeong H., Tran P.K.L., Malhotra D., Jeong K.U., Kim N.H., Lee J.H. (2022). Recent engineering advances in nanocatalysts for NH_3_-to-H_2_ conversion technologies. Nano Energy.

[12] Juangsa F.B., Irhamna A.R., Aziz M. (2021). Production of ammonia as potential hydrogen carrier: Review on thermochemical and electrochemical processes. Int. J. Hydrogen Energy.

[13] Peters S., Abdel-Mageed A.M., Wohlrab S. (2022). Thermocatalytic Ammonia Decomposition—Status and Current Research Demands for a Carbon-Free Hydrogen Fuel Technology. ChemCatChem.

[14] Li S., Miao P., Zhang Y., Wu J., Zhang B., Du Y., Han X., Sun J., Xu P. (2021). Recent Advances in Plasmonic Nanostructures for Enhanced Photocatalysis and Electrocatalysis. Adv. Mater..

[15] Zhang S., He Z., Li X., Zhang J., Zang Q., Wang S. (2020). Building heterogeneous nanostructures for photocatalytic ammonia decomposition. Nanoscale Adv..

[16] Dong B.X., Ichikawa T., Hanada N., Hino S., Kojima Y. (2011). Liquid ammonia electrolysis by platinum electrodes. J. Alloys Compd..

[17] Little D.J., Smith I.I.I.M.R., Hamann T.W. (2015). Electrolysis of liquid ammonia for hydrogen generation. Energy Environ. Sci..

[18] Lee D.H., Kang H., Kim Y., Song H., Lee H., Choi J., Kim K.-T., Song Y.-H. (2023). Plasma-assisted hydrogen generation: A mechanistic review. Fuel Process. Technol..

[19] Mohammadi Z., Sharifnia S., Shavisi Y. (2016). Photocatalytic degradation of aqueous ammonia by using TiO2ZnO/LECA hybrid photocatalyst. Mater. Chem. Phys..

[20] Shiraishi Y., Toi S., Ichikawa S., Hirai T. (2020). Photocatalytic NH_3_ Splitting on TiO_2_ Particles Decorated with Pt–Au Bimetallic Alloy Nanoparticles. ACS Appl. Nano Mater..

[21] Gao Y., Hu E., Yi Y., Yin G., Huang Z. (2023). Plasma-assisted low temperature ammonia decomposition on 3d transition metal (Fe, Co and Ni) doped CeO_2_ catalysts: Synergetic effect of morphology and co-doping. Fuel Process. Technol..

[22] Yi Y., Wang L., Guo Y., Sun S., Guo H. (2018). Plasma-Assisted ammonia decomposition over Fe–Ni alloy catalysts for CO_x_-Free hydrogen. AIChE J..

[23] Le T.A., Kim Y., Kim H.W., Lee S.-U., Kim J.-R., Kim T.-W., Lee Y.-J., Chae H.-J. (2021). Ru-supported lanthania-ceria composite as an efficient catalyst for CO_x_-free H_2_ production from ammonia decomposition. Appl. Catal. B Environ..

[24] Shavisi Y., Sharifnia S., Mohamadi Z. (2016). Solar-light-harvesting degradation of aqueous ammonia by CuO/ZnO immobilized on pottery plate: Linear kinetic modeling for adsorption and photocatalysis process. J. Environ. Chem. Eng..

[25] Gu Y.-Q., Jin Z., Zhang H., Xu R.-J., Zheng M.-J., Guo Y.-M., Song Q.-S., Jia C.-J. (2015). Transition metal nanoparticles dispersed in an alumina matrix as active and stable catalysts for COx-free hydrogen production from ammonia. J. Mater. Chem. A.

[26] Alturaifi S.A., Mathieu O., Petersen E.L. (2022). An experimental and modeling study of ammonia pyrolysis. Combust. Flame.

[27] Hayashi F., Toda Y., Kanie Y., Kitano M., Inoue Y., Yokoyama T., Hara M., Hosono H. (2013). Ammonia decomposition by ruthenium nanoparticles loaded on inorganic electride C12A7:e−. Chem. Sci..

[28] Khan W.U., Alasiri H.S., Ali S.A., Hossain M.M. (2022). Recent Advances in Bimetallic Catalysts for Hydrogen Production from Ammonia. Chem. Rec..

[29] Ao R., Lu R., Leng G., Zhu Y., Yan F., Yu Q. (2023). A Review on Numerical Simulation of Hydrogen Production from Ammonia Decomposition. Energies.

[30] Wang Z., Cai Z., Wei Z. (2019). Highly Active Ruthenium Catalyst Supported on Barium Hexaaluminate for Ammonia Decomposition to CO_x_-Free Hydrogen. ACS Sustain. Chem. Eng..

[31] Chen C., Wu K., Ren H., Zhou C., Luo Y., Lin L., Au C., Jiang L. (2021). Ru-Based Catalysts for Ammonia Decomposition: A Mini-Review. Energy Fuels.

[32] Zhiqiang F., Ziqing W., Dexing L., Jianxin L., Lingzhi Y., Qin W., Zhong W. (2022). Catalytic ammonia decomposition to CO_x_-free hydrogen over ruthenium catalyst supported on alkali silicates. Fuel.

[33] Wang Y., Mao X., Yang J., Wang J., Guan W., Chen J., Han B., Tian Z. (2022). One-step synthesis of Ni/yttrium-doped barium zirconates catalyst for on-site hydrogen production from NH_3_ decomposition. Int. J. Hydrogen Energy.

[34] Qiu Y., Fu E., Gong F., Xiao R. (2022). Catalyst support effect on ammonia decomposition over Ni/MgAl_2_O_4_ towards hydrogen production. Int. J. Hydrogen Energy.

[35] Kocer T., Saraç-Öztuna F.E., Kurtoğlu-Öztulum S.F., Unal U., Uzun A. (2022). Effect of Nickel Precursor on the Catalytic Performance of Graphene Aerogel-Supported Nickel Nanoparticles for the Production of CO_x_-free Hydrogen by Ammonia Decomposition. Energy Technol..

[36] Cui H.-Z., Gu Y.-Q., He X.-X., Wei S., Jin Z., Jia C.-J., Song Q.-S. (2016). Iron-based composite nanostructure catalysts used to produce CO_x_-free hydrogen from ammonia. Sci. Bull..

[37] Martirez J.M.P., Carter E.A. (2022). First-Principles Insights into the Thermocatalytic Cracking of Ammonia-Hydrogen Blends on Fe(110): 1. Thermodynamics. J. Phys. Chem. C.

[38] Lu B., Li L., Ren M., Liu Y., Zhang Y., Xu X., Wang X., Qiu H. (2022). Ammonia decomposition over iron-based catalyst: Exploring the hidden active phase. Appl. Catal. B Environ..

[39] Hu X.-C., Wang W.-W., Jin Z., Wang X., Si R., Jia C.-J. (2019). Transition metal nanoparticles supported La-promoted MgO as catalysts for hydrogen production via catalytic decomposition of ammonia. J. Energy Chem..

[40] Maslova V., Fourré E., Veryasov G., Nesterenko N., Grishin A., Louste C., Nassar M., Guignard N., Arrii S., Batiot-Dupeyrat C. (2023). Ammonia Decomposition in Electric Field over Ce-based Materials. ChemCatChem.

[41] Podila S., Driss H., Ali A.M., Al-Zahrani A.A., Daous M.A. (2022). Influence of Ce substitution in LaMO_3_ (M = Co/Ni) perovskites for CO_x_-free hydrogen production from ammonia decomposition. Arab. J. Chem..

[42] Huang C., Li H., Yang J., Wang C., Hu F., Wang X., Lu Z.-H., Feng G., Zhang R. (2019). Ce_0.6_Zr_0.3_Y_0.1_O_2_ solid solutions-supported Ni-Co bimetal nanocatalysts for NH_3_ decomposition. Appl. Surf. Sci..

[43] Sima D., Wu H., Tian K., Xie S., Foo J.J., Li S., Wang D., Ye Y., Zheng Z., Liu Y.-Q. (2020). Enhanced low temperature catalytic activity of Ni/Al–Ce_0.8_Zr_0.2_O_2_ for hydrogen production from ammonia decomposition. Int. J. Hydrogen Energy.

[44] Xie P., Yao Y., Huang Z., Liu Z., Zhang J., Li T., Wang G., Shahbazian-Yassar R., Hu L., Wang C. (2019). Highly efficient decomposition of ammonia using high-entropy alloy catalysts. Nat. Commun..

[45] Sayas S., Morlanés N., Katikaneni S.P., Harale A., Solami B., Gascon J. (2020). High pressure ammonia decomposition on Ru–K/CaO catalysts. Catal. Sci. Technol..

[46] Wu Z.-W., Li X., Qin Y.-H., Deng L., Wang C.-W., Jiang X. (2020). Ammonia decomposition over SiO_2_-supported Ni–Co bimetallic catalyst for CO_x_-free hydrogen generation. Int. J. Hydrogen Energy.

[47] Simonsen S.B., Chakraborty D., Chorkendorff I., Dahl S. (2012). Alloyed Ni-Fe nanoparticles as catalysts for NH_3_ decomposition. Appl. Catal. A Gen..

[48] Fu E., Qiu Y., Lu H., Wang S., Liu L., Feng H., Yang Y., Wu Z., Xie Y., Gong F. (2021). Enhanced NH_3_ decomposition for H_2_ production over bimetallic M(M=Co, Fe, Cu)Ni/Al_2_O_3_. Fuel Process. Technol..

[49] Tabassum H., Mukherjee S., Chen J., Holiharimanana D., Karakalos S., Yang X., Hwang S., Zhang T., Lu B., Chen M. (2022). Hydrogen generation via ammonia decomposition on highly efficient and stable Ru-free catalysts: Approaching complete conversion at 450 °C. Energy Environ. Sci..

[50] Nagaoka K., Eboshi T., Takeishi Y., Tasaki R., Honda K., Imamura K., Sato K. (2017). Carbon-free H_2_ production from ammonia triggered at room temperature with an acidic RuO_2_/gamma-Al_2_O_3_ catalyst. Sci. Adv..

[51] Navascués P., Obrero-Pérez J.M., Cotrino J., González-Elipe A.R., Gómez-Ramírez A. (2020). Unraveling Discharge and Surface Mechanisms in Plasma-Assisted Ammonia Reactions. ACS Sustain. Chem. Eng..

[52] Wang L., Yi Y., Guo Y., Zhao Y., Zhang J., Guo H. (2017). Synergy of DBD plasma and Fe-based catalyst in NH_3_ decomposition: Plasma enhancing adsorption step. Plasma Process. Polym..

[53] Akiyama M., Aihara K., Sawaguchi T., Matsukata M., Iwamoto M. (2018). Ammonia decomposition to clean hydrogen using non-thermal atmospheric-pressure plasma. Int. J. Hydrogen Energy.

[54] El-Shafie M., Kambara S., Hayakawa Y. (2020). Alumina particle size effect on H2 production from ammonia decomposition by DBD plasma. Energy Rep..

[55] Wang L., Zhao Y., Liu C., Gong W., Guo H. (2013). Plasma driven ammonia decomposition on a Fe-catalyst: Eliminating surface nitrogen poisoning. Chem. Commun..

[56] Wang L., Yi Y., Zhao Y., Zhang R., Zhang J., Guo H. (2015). NH_3_ Decomposition for H_2_ Generation: Effects of Cheap Metals and Supports on Plasma–Catalyst Synergy. ACS Catal..

[57] Chen G., Qu J., Cheah P., Cao D., Zhao Y., Xiang Y. (2022). Size-Dependent Activity of Iron Nanoparticles in Both Thermal and Plasma Driven Catalytic Ammonia Decomposition. Ind. Eng. Chem. Res..

[58] El-Shafie M., Kambara S., Hayakawa Y. (2021). Energy and exergy analysis of hydrogen production from ammonia decomposition systems using non-thermal plasma. Int. J. Hydrogen Energy.

[59] Lin Q.F., Jiang Y.M., Liu C.Z., Chen L.W., Zhang W.J., Ding J., Li J.G. (2021). Instantaneous hydrogen production from ammonia by non-thermal arc plasma combining with catalyst. Energy Rep..

[60] El-Shafie M., Kambara S., Hayakawa Y. (2021). Plasma-enhanced catalytic ammonia decomposition over ruthenium (Ru/Al_2_O_3_) and soda glass (SiO_2_) materials. J. Energy Inst..

[61] Andersen J.A., Christensen J.M., Østberg M., Bogaerts A., Jensen A.D. (2022). Plasma-catalytic ammonia decomposition using a packed-bed dielectric barrier discharge reactor. Int. J. Hydrogen Energy.

[62] Bang S., Snoeckx R., Cha M.S. (2023). Kinetic Study for Plasma Assisted Cracking of NH_3_: Approaches and Challenges. J. Phys. Chem. A.

[63] Hanada N., Hino S., Ichikawa T., Suzuki H., Takai K., Kojima Y. (2010). Hydrogen generation by electrolysis of liquid ammonia. Chem. Commun. (Camb).

[64] Yang Y., Kim J., Jo H., Seong A., Lee M., Min H.-K., Seo M.-g., Choi Y., Kim G. (2021). A rigorous electrochemical ammonia electrolysis protocol with in operando quantitative analysis. J. Mater. Chem. A.

[65] Goshome K., Yamada T., Miyaoka H., Ichikawa T., Kojima Y. (2016). High compressed hydrogen production via direct electrolysis of liquid ammonia. Int. J. Hydrogen Energy.

[66] Akagi N., Hori K., Sugime H., Noda S., Hanada N. (2022). Systematic investigation of anode catalysts for liquid ammonia electrolysis. J. Catal..

[67] Ponikvar Ž., Likozar B., Gyergyek S. (2022). Electrification of Catalytic Ammonia Production and Decomposition Reactions: From Resistance, Induction, and Dielectric Reactor Heating to Electrolysis. ACS Appl. Energy Mater..

[68] Jiang M., Zhu D., Zhao X. (2014). Electrolysis of ammonia for hydrogen production catalyzed by Pt and Pt-Ir deposited on nickel foam. J. Energy Chem..

[69] Dong B.-X., Tian H., Wu Y.-C., Bu F.-Y., Liu W.-L., Teng Y.-L., Diao G.-W. (2016). Improved electrolysis of liquid ammonia for hydrogen generation via ammonium salt electrolyte and Pt/Rh/Ir electrocatalysts. Int. J. Hydrogen Energy.

[70] Little D.J., Edwards D.O., Smith M.R., Hamann T.W. (2017). As Precious as Platinum: Iron Nitride for Electrocatalytic Oxidation of Liquid Ammonia. ACS Appl. Mater. Interfaces.

[71] Wu M., Du J., Tao C., Liu Z., Li Y. (2019). A tri-functionalised PtSnO_x_-based electrocatalyst for hydrogen generation via ammonia decomposition under native pH conditions. J. Colloid Interface Sci..

[72] Zhang S., Zhao Y., Yan L., Jiang H., Yang X., Wang Y., Song H., Zhao X. (2021). Electrochemical ammonia oxidation reaction on defect-rich TiO nanofibers: Experimental and theoretical studies. Int. J. Hydrogen Energy.

[73] Zhang S., Yan L., Jiang H., Yang L., Zhao Y., Yang X., Wang Y., Shen J., Zhao X. (2022). Facile Fabrication of a Foamed Ag_3_CuS_2_ Film as an Efficient Self-Supporting Electrocatalyst for Ammonia Electrolysis Producing Hydrogen. ACS Appl. Mater. Interfaces.

[74] Dong Y., Bai Z., Liu R., Zhu T. (2007). Preparation of fibrous TiO_2_ photocatalyst and its optimization towards the decomposition of indoor ammonia under illumination. Catal. Today.

[75] Fuku K., Kamegawa T., Mori K., Yamashita H. (2012). Highly dispersed platinum nanoparticles on TiO_2_ prepared by using the microwave-assisted deposition method: An efficient photocatalyst for the formation of H_2_ and N_2_ from aqueous NH_3_. Chem. Asian J..

[76] Kominami H., Nishimune H., Ohta Y., Arakawa Y., Inaba T. (2012). Photocatalytic hydrogen formation from ammonia and methyl amine in an aqueous suspension of metal-loaded titanium(IV) oxide particles. Appl. Catal. B Environ..

[77] Yuzawa H., Mori T., Itoh H., Yoshida H. (2012). Reaction Mechanism of Ammonia Decomposition to Nitrogen and Hydrogen over Metal Loaded Titanium Oxide Photocatalyst. J. Phys. Chem. C.

[78] Reli M., Edelmannová M., Šihor M., Praus P., Svoboda L., Mamulová K.K., Otoupalíková H., Čapek L., Hospodková A., Obalová L. (2015). Photocatalytic H_2_ generation from aqueous ammonia solution using ZnO photocatalysts prepared by different methods. Int. J. Hydrogen Energy.

[79] Iwase A., Ii K., Kudo A. (2018). Decomposition of an aqueous ammonia solution as a photon energy conversion reaction using a Ru-loaded ZnS photocatalyst. Chem. Commun..

[80] Jung S.-C., Chung K.-H., Choi J., Park Y.-K., Kim S.-J., Kim B.-J., Lee H. (2022). Photocatalytic hydrogen production using liquid phase plasma from ammonia water over metal ion-doped TiO_2_ photocatalysts. Catal. Today.

[81] Wu Z., Ambrožová N., Eftekhari E., Aravindakshan N., Wang W., Wang Q., Zhang S., Kočí K., Li Q. (2019). Photocatalytic H_2_ generation from aqueous ammonia solution using TiO_2_ nanowires-intercalated reduced graphene oxide composite membrane under low power UV light. Emergent Mater..

[82] Zaman S.F., Jolaoso L.A., Podila S., Al-Zahrani A.A., Alhamed Y.A., Driss H., Daous M.M., Petrov L. (2018). Ammonia decomposition over citric acid chelated γ-Mo2N and Ni2Mo3N catalysts. Int. J. Hydrogen Energy.

[83] Utsunomiya A., Okemoto A., Nishino Y., Kitagawa K., Kobayashi H., Taniya K., Ichihashi Y., Nishiyama S. (2017). Mechanistic study of reaction mechanism on ammonia photodecomposition over Ni/TiO_2_ photocatalysts. Appl. Catal. B Environ..

[84] Abdul Razak S., Mahadi A.H., Thotagamuge R., Prasetyoko D., Bahruji H. (2022). Photocatalytic Hydrogen Gas Production from NH3 and Alkylamine: Route to Zero Carbon Emission Energy. Catal. Lett..

[85] Yuan Y., Zhou L., Robatjazi H., Bao J.L., Zhou J., Bayles A., Yuan L., Lou M., Lou M., Khatiwada S. (2022). Earth-abundant photocatalyst for H_2_ generation from NH_3_ with light-emitting diode illumination. Science.

[86] Leung K.C., Tan E., Li G., Ng B.K.Y., Ho P.-L., Lebedev K., Tsang S.C.E. (2023). Metal-loaded zeolites in ammonia decomposition catalysis. Faraday Discuss.

[87] Kim H.B., Park E.D. (2023). Ammonia decomposition over Ru catalysts supported on alumina with different crystalline phases. Catal. Today.

[88] Pinzón M., Avilés-García O., de la Osa A.R., de Lucas-Consuegra A., Sánchez P., Romero A. (2022). New catalysts based on reduced graphene oxide for hydrogen production from ammonia decomposition. Sustain. Chem. Pharm..

[89] Lu X., Zhang J., Chen W.K., Roldan A. (2021). Kinetic and mechanistic analysis of NH_3_ decomposition on Ru(0001), Ru(111) and Ir(111) surfaces. Nanoscale Adv..

[90] Armenise S., Cazaña F., Monzón A., García-Bordejé E. (2018). In situ generation of CO_x_-free H_2_ by catalytic ammonia decomposition over Ru-Al-monoliths. Fuel.

[91] García-García F.R., Guerrero-Ruiz A., Rodríguez-Ramos I. (2009). Role of B5-Type Sites in Ru Catalysts used for the NH_3_ Decomposition Reaction. Top. Catal..

[92] Feng J., Liu L., Ju X., Wang J., Zhang X., He T., Chen P. (2021). Highly Dispersed Ruthenium Nanoparticles on Y_2_O_3_ as Superior Catalyst for Ammonia Decomposition. ChemCatChem.

[93] Cha J., Lee T., Lee Y.-J., Jeong H., Jo Y.S., Kim Y., Nam S.W., Han J., Lee K.B., Yoon C.W. (2021). Highly monodisperse sub-nanometer and nanometer Ru particles confined in alkali-exchanged zeolite Y for ammonia decomposition. Appl. Catal. B Environ..

[94] Hu X.-C., Fu X.-P., Wang W.-W., Wang X., Wu K., Si R., Ma C., Jia C.-J., Yan C.-H. (2020). Ceria-supported ruthenium clusters transforming from isolated single atoms for hydrogen production via decomposition of ammonia. Appl. Catal. B Environ..

[95] Jeon N., Kim S., Tayal A., Oh J., Yoon W., Kim W.B., Yun Y. (2022). Y-Doped BaCeO_3_ Perovskite-Supported Ru Catalysts for CO_x_-Free Hydrogen Production from Ammonia: Effect of Strong Metal–Support Interactions. ACS Sustain. Chem. Eng..

[96] Im Y., Muroyama H., Matsui T., Eguchi K. (2022). Investigation on catalytic performance and desorption behaviors of ruthenium catalysts supported on rare-earth oxides for NH_3_ decomposition. Int. J. Hydrogen Energy.

[97] Cao C.-F., Wu K., Zhou C., Yao Y.-H., Luo Y., Chen C.-Q., Lin L., Jiang L. (2022). Electronic metal-support interaction enhanced ammonia decomposition efficiency of perovskite oxide supported ruthenium. Chem. Eng. Sci..

[98] Zhang X., Liu L., Feng J., Ju X., Wang J., He T., Chen P. (2021). Metal–support interaction-modulated catalytic activity of Ru nanoparticles on Sm_2_O_3_ for efficient ammonia decomposition. Catal. Sci. Technol..

[99] Yamazaki K., Matsumoto M., Ishikawa M., Sato A. (2023). NH_3_ decomposition over Ru/CeO_2_-PrO_x_ catalyst under high space velocity conditions for an on-site H_2_ fueling station. Appl. Catal. B Environ..

[100] Obata K., Kishishita K., Okemoto A., Taniya K., Ichihashi Y., Nishiyama S. (2014). Photocatalytic decomposition of NH_3_ over TiO_2_ catalysts doped with Fe. Appl. Catal. B Environ..

[101] Bell T.E., Torrente-Murciano L. (2016). H_2_ Production via Ammonia Decomposition Using Non-Noble Metal Catalysts: A Review. Top. Catal..

[102] Zhang J., Xu H., Li W. (2005). Kinetic study of NH_3_ decomposition over Ni nanoparticles: The role of La promoter, structure sensitivity and compensation effect. Appl. Catal. A Gen..

[103] Hu Z.-P., Weng C.-C., Yuan G.-G., Lv X.-W., Yuan Z.-Y. (2018). Ni nanoparticles supported on mica for efficient decomposition of ammonia to CO_x_-free hydrogen. Int. J. Hydrogen Energy.

[104] Deng L., Lin H., Liu X., Xu J., Zhou Z., Xu M. (2021). Nickel nanoparticles derived from the direct thermal reduction of Ni-containing Ca–Al layered double hydroxides for hydrogen generation via ammonia decomposition. Int. J. Hydrogen Energy.

[105] Zhang H., Alhamed Y.A., Kojima Y., Al-Zahrani A.A., Miyaoka H., Petrov L.A. (2014). Structure and catalytic properties of Ni/MWCNTs and Ni/AC catalysts for hydrogen production via ammonia decomposition. Int. J. Hydrogen Energy.

[106] Wu K., Cao C.-F., Zhou C., Luo Y., Chen C.-Q., Lin L., Au C., Jiang L. (2021). Engineering of Ce^3+^-O-Ni structures enriched with oxygen vacancies via Zr doping for effective generation of hydrogen from ammonia. Chem. Eng. Sci..

[107] Zhou C., Wu K., Huang H., Cao C.-F., Luo Y., Chen C.-Q., Lin L., Au C., Jiang L. (2021). Spatial Confinement of Electron-Rich Ni Nanoparticles for Efficient Ammonia Decomposition to Hydrogen Production. ACS Catal..

[108] Chen C., Fan X., Zhou C., Lin L., Luo Y., Au C., Cai G., Wang X., Jiang L. (2023). Hydrogen production from ammonia decomposition over Ni/CeO_2_ catalyst: Effect of CeO_2_ morphology. J. Rare Earths.

[109] Li Y., Guan Q., Huang G., Yuan D., Xie F., Li K., Zhang Z., San X., Ye J. (2022). Low Temperature Thermal and Solar Heating Carbon-Free Hydrogen Production from Ammonia Using Nickel Single Atom Catalysts. Adv. Energy Mater..

[110] Okura K., Okanishi T., Muroyama H., Matsui T., Eguchi K. (2016). Ammonia Decomposition over Nickel Catalysts Supported on Rare-Earth Oxides for the On-Site Generation of Hydrogen. ChemCatChem.

[111] Okura K., Okanishi T., Muroyama H., Matsui T., Eguchi K. (2016). Additive effect of alkaline earth metals on ammonia decomposition reaction over Ni/Y_2_O_3_ catalysts. RSC Adv..

[112] Gu Y., Ma Y., Long Z., Zhao S., Wang Y., Zhang W. (2021). One-pot synthesis of supported Ni@Al_2_O_3_ catalysts with uniform small-sized Ni for hydrogen generation via ammonia decomposition. Int. J. Hydrogen Energy.

[113] Okura K., Okanishi T., Muroyama H., Matsui T., Eguchi K. (2015). Promotion effect of rare-earth elements on the catalytic decomposition of ammonia over Ni/Al_2_O_3_ catalyst. Appl. Catal. A Gen..

[114] Do Q.C., Kim Y., Le T.A., Kim G.J., Kim J.-R., Kim T.-W., Lee Y.-J., Chae H.-J. (2022). Facile one-pot synthesis of Ni-based catalysts by cation-anion double hydrolysis method as highly active Ru-free catalysts for green H_2_ production via NH_3_ decomposition. Appl. Catal. B Environ..

[115] Yu Y., Gan Y.-M., Huang C., Lu Z.-H., Wang X., Zhang R., Feng G. (2020). Ni/La_2_O_3_ and Ni/MgO–La_2_O_3_ catalysts for the decomposition of NH_3_ into hydrogen. Int. J. Hydrogen Energy.

[116] Varisli D., Kaykac N.G. (2012). CO_x_ free hydrogen production over cobalt incorporated silicate structured mesoporous catalysts. Appl. Catal. B Environ..

[117] Lendzion-Bielun Z., Narkiewicz U., Arabczyk W. (2013). Cobalt-based Catalysts for Ammonia Decomposition. Materials.

[118] Pinzón M., Romero A., de Lucas-Consuegra A., de la Osa A.R., Sánchez P. (2022). CO_x_-free hydrogen production from ammonia at low temperature using Co/SiC catalyst: Effect of promoter. Catal. Today.

[119] Zhang H., Alhamed Y.A., Chu W., Ye Z., AlZahrani A., Petrov L. (2013). Controlling Co-support interaction in Co/MWCNTs catalysts and catalytic performance for hydrogen production via NH_3_ decomposition. Appl. Catal. A Gen..

[120] Duan X., Ji J., Qian G., Fan C., Zhu Y., Zhou X., Chen D., Yuan W. (2012). Ammonia decomposition on Fe(1 1 0), Co(1 1 1) and Ni(1 1 1) surfaces: A density functional theory study. J. Mol. Catal. A Chem..

[121] Czekajło Ł., Lendzion-Bieluń Z. (2016). Effect of preparation conditions and promoters on the structure and activity of the ammonia decomposition reaction catalyst based on nanocrystalline cobalt. Chem. Eng. J..

[122] Varisli D., Kaykac N.G. (2016). Hydrogen from ammonia over cobalt incorporated silicate structured catalysts prepared using different cobalt salts. Int. J. Hydrogen Energy.

[123] Huang C., Yu Y., Tang X., Liu Z., Zhang J., Ye C., Ye Y., Zhang R. (2020). Hydrogen generation by ammonia decomposition over Co/CeO_2_ catalyst: Influence of support morphologies. Appl. Surf. Sci..

[124] Yu P., Wu H., Guo J., Wang P., Chang F., Gao W., Zhang W., Liu L., Chen P. (2020). Effect of BaNH, CaNH, Mg_3_N_2_ on the activity of Co in NH_3_ decomposition catalysis. J. Energy Chem..

[125] Li G., Zhang H., Yu X., Lei Z., Yin F., He X. (2022). Highly efficient Co/NC catalyst derived from ZIF-67 for hydrogen generation through ammonia decomposition. Int. J. Hydrogen Energy.

[126] Lanzani G., Laasonen K. (2010). NH_3_ adsorption and dissociation on a nanosized iron cluster. Int. J. Hydrogen Energy.

[127] Othman N.E.F., Salleh H.M., Purwanto H. (2016). Utilization of Low-grade Iron Ore in Ammonia Decomposition. Procedia Chem..

[128] Lucentini I., García Colli G., Luzi C., Serrano I., Soler L., Divins N.J., Martínez O.M., Llorca J. (2022). Modelling and simulation of catalytic ammonia decomposition over Ni-Ru deposited on 3D-printed CeO_2_. Chem. Eng. J..

[129] Lucentini I., García Colli G., Luzi C.D., Serrano I., Martínez O.M., Llorca J. (2021). Catalytic ammonia decomposition over Ni-Ru supported on CeO_2_ for hydrogen production: Effect of metal loading and kinetic analysis. Appl. Catal. B Environ..

[130] Lucentini I., Casanovas A., Llorca J. (2019). Catalytic ammonia decomposition for hydrogen production on Ni, Ru and Ni-Ru supported on CeO_2_. Int. J. Hydrogen Energy.

[131] Li H., Guo L., Qu J., Fang X., Fu Y., Duan J., Wang W., Li C. (2023). Co-Ni supported yttrium oxide material as a catalyst for ammonia decomposition to CO_x_-free hydrogen. Int. J. Hydrogen Energy.

[132] He H., Jiang H., Yang F., Liu J., Zhang W., Jin M., Li Z. (2023). Bimetallic Ni_x_Co_10−x_/CeO_2_ as highly active catalysts to enhance mid-temperature ammonia decomposition: Kinetics and synergies. Int. J. Hydrogen Energy.

[133] Silva H., Nielsen M.G., Fiordaliso E.M., Damsgaard C.D., Gundlach C., Kasama T., Chorkendorff I.B., Chakraborty D. (2015). Synthesis and characterization of Fe–Ni/ɣ-Al_2_O_3_ egg-shell catalyst for H_2_ generation by ammonia decomposition. Appl. Catal. A Gen..

[134] Hansgen D.A., Vlachos D.G., Chen J.G. (2010). Using first principles to predict bimetallic catalysts for the ammonia decomposition reaction. Nat. Chem..

[135] Jiang K., Li K., Liu Y.-Q., Lin S., Wang Z., Wang D., Ye Y. (2022). Nickel-cobalt nitride nanoneedle supported on nickel foam as an efficient electrocatalyst for hydrogen generation from ammonia electrolysis. Electrochim. Acta.

[136] Huo L., Han X., Zhang L., Liu B., Gao R., Cao B., Wang W.-W., Jia C.-J., Liu K., Liu J. (2021). Spatial confinement and electron transfer moderating Mo-N bond strength for superior ammonia decomposition catalysis. Appl. Catal. B Environ..

[137] Xu J., Yan H., Jin Z., Jia C.J. (2019). Facile Synthesis of Stable MO_2_N Nanobelts with High Catalytic Activity for Ammonia Decomposition. Chin. J. Chem..

[138] Podila S., Zaman S.F., Driss H., Alhamed Y.A., Al-Zahrani A.A., Petrov L.A. (2016). Hydrogen production by ammonia decomposition using high surface area Mo_2_N and Co_3_Mo_3_N catalysts. Catal. Sci. Technol..

[139] Guo J., Wang P., Wu G., Wu A., Hu D., Xiong Z., Wang J., Yu P., Chang F., Chen Z. (2015). Lithium imide synergy with 3d transition-metal nitrides leading to unprecedented catalytic activities for ammonia decomposition. Angew. Chem. Int. Ed. Engl..

[140] Jolaoso L.A., Zaman S.F., Podila S., Driss H., Al-Zahrani A.A., Daous M.A., Petrov L. (2018). Ammonia decomposition over citric acid induced γ-Mo_2_N and Co_3_Mo_3_N catalysts. Int. J. Hydrogen Energy.

[141] Srifa A., Okura K., Okanishi T., Muroyama H., Matsui T., Eguchi K. (2016). CO_x_-free hydrogen production via ammonia decomposition over molybdenum nitride-based catalysts. Catal. Sci. Technol..

[142] Srifa A., Okura K., Okanishi T., Muroyama H., Matsui T., Eguchi K. (2017). Hydrogen production by ammonia decomposition over Cs-modified Co_3_Mo_3_N catalysts. Appl. Catal. B Environ..

[143] Kraupner A., Markus A., Palkovits R., Schlicht K., Giordano C. (2010). Mesoporous Fe_3_C sponges as magnetic supports and as heterogeneous catalyst. J. Mater. Chem..

[144] Santhana Krishnan P., Neelaveni M., Tamizhdurai P., Mythily M., Krishna Mohan S., Mangesh V.L., Shanthi K. (2020). CO_x_-free hydrogen generation via decomposition of ammonia over al, Ti and Zr−Laponite supported MoS_2_ catalysts. Int. J. Hydrogen Energy.

